# The pyrin inflammasome, a leading actor in pediatric autoinflammatory diseases

**DOI:** 10.3389/fimmu.2023.1341680

**Published:** 2024-01-05

**Authors:** Saverio La Bella, Armando Di Ludovico, Giulia Di Donato, Ozge Basaran, Seza Ozen, Marco Gattorno, Francesco Chiarelli, Luciana Breda

**Affiliations:** ^1^ Department of Pediatrics, "G. D'Annunzio" University of Chieti, Chieti, Italy; ^2^ Department of Pediatrics, Division of Rheumatology, Hacettepe University Faculty of Medicine, Ankara, Türkiye; ^3^ UOC Rheumatology and Autoinflammatory Diseases, IRCCS Istituto Giannina Gaslini, Genova, Italy

**Keywords:** pyrin, inflammasome, familial Mediterranean fever, FMF, autoinflammatory diseases, IL-1, gasdermin D

## Abstract

The activation of the pyrin inflammasome represents a highly intriguing mechanism employed by the innate immune system to effectively counteract pathogenic agents. Despite its key role in innate immunity, pyrin has also garnered significant attention due to its association with a range of autoinflammatory diseases (AIDs) including familial Mediterranean fever caused by disruption of the *MEFV* gene, or in other genes involved in its complex regulation mechanisms. Pyrin activation is strictly dependent on homeostasis-altering molecular processes, mostly consisting of the disruption of the small Ras Homolog Family Member A (RhoA) GTPases by pathogen toxins. The downstream pathways are regulated by the phosphorylation of specific pyrin residues by the kinases PKN1/2 and the binding of the chaperone 14-3-3. Furthermore, a key role in pyrin activation is played by the cytoskeleton and gasdermin D, which is responsible for membrane pores in the context of pyroptosis. In addition, recent evidence has highlighted the role of steroid hormone catabolites and alarmins S100A8/A9 and S100A12 in pyrin-dependent inflammation. The aim of this article is to offer a comprehensive overview of the most recent evidence on the pyrin inflammasome and its molecular pathways to better understand the pathogenesis behind the significant group of pyrin-related AIDs.

## 1 Introduction

Autoinflammatory diseases (AIDs) are a large group of inherited disorders characterized by a dysregulation of the innate immune system; the dysregulation is associated with sterile inflammation mostly caused by an overproduction of proinflammatory cytokines (1, 2). AIDs usually present in infancy or childhood with systemic inflammation causing recurrent fever and multiorgan involvement, including the skin, serosal membranes, gastrointestinal tube, central nervous system, and other tissues (1, 3). Due to their clinical heterogeneity and rarity, these diseases are frequently misinterpreted, resulting in delayed diagnosis and treatment ^1^. These disorders can be caused by a large variety of pathogenic processes, including dysregulated inflammasome-mediated cytokine production (4–6). AIDs are characterized by a strong genetic background, and those resulting from mutations in single genes are commonly known as “monogenic AIDs” ^1^. The pyrin inflammasome is a leading actor in the fascinating field of autoinflammation and plays a key role in a substantial number of monogenic AIDs, including familial Mediterranean fever (FMF) and mevalonate kinase deficiency (MKD) (**Table 1**). FMF is caused by point pathogenic variants in the pyrin-encoding *MEFV* gene and was the first AID to be described as a distinct entity in 1945, although genetically characterized in 1997 (7, 8). Over the past two decades, the understanding of the genetic background and pathogenic mechanisms underlying the pyrin inflammasome has made great progress. The growing knowledge acquired about *MEFV* structure and pyrin’s regulatory mechanisms has allowed a better understanding of the underlying innate pathways, opening new perspectives for novel targeted therapies (6, 9). The aim of this review is to provide a thorough and all-encompassing synopsis of the latest findings concerning the pyrin inflammasome and its associated regulatory mechanisms, with a particular focus on the correlation between molecular pathways and clinical manifestations. In order to provide a comprehensive understanding of this intriguing innate immunity actor, the most recent knowledge on the *MEFV* gene and its regulatory pathways, the clinical features associated to the diverse pathogenic variants, and the critical interactions of the various pyrin domains has been elaborated and expanded.

**Table 1:** Autoinflammatory diseases related to disfunctions of pyrin molecular pathways.

Disease	Gene (pathogenic variants)	Gene locus	Protein	Pathogenesis	Main clinical manifestations	Inheritance
***ARPC1B*-related AID**	*ARPC1B* (p.V91Wfs*30, p.A105V, p.A238T)	7q22.1	Actin-related protein 2/3 complex subunit 1B (ARPC1B)	Not well characterized. F-actin branching defects due to ARPC1B deficiency.	Platelet abnormalities due to disrupted spreading, lymphoproliferation, immune deficiency, eosinophilia, and systemic autoinflammation	AR
**FMF**	*MEFV*	16p13.3	Pyrin	Gain of function mutations causing poor affinity to regulatory proteins (PKN1, PKN2, 14-3-3) and constitutive activation of pyrin inflammasome	Fever (12-72 hours), serositis (abdominal pain, chest pain), non-erosive acute arthritis, erysipelas-like rash	AR/AD
**MKD** **PK**	*MVK*	12q24.11	Mevalonate kinase (MVK)	Reduced prenylation of proteins, necessary for RhoA activation and PI3K-mediated inhibition of pyrin inflammasome	Early-onset (less than 1 year), fever (3-7 days), GI symptoms, arthromyalgia or arthritis, maculo-papular or urticarial rash, aphthous stomatitis, hepatosplenomegaly, cervical adenopathy	AR
**NOCARH**	*CDC42* (p.C188Y, p.R186C, p.*192C*24)	1p36.12	Cell division control protein 42 homolog (CDC42)	Increased secretion of IL-18 due to cytoskeletal abnormalities	Neonatal onset of cytopenia, rash, and hemophagocytes due to systemic autoinflammation	Unknown
**PAAND**	*MEFV* (p.S242R p.E244K)	16p13.3	Pyrin	Loss of pyrin inhibition by 14-3-3 protein	Fever, neutrophilic dermatosis, acne, pyoderma gangrenosum, cutaneous abscesses	AD
**PAPA**	*PSTPIP1* (p.A230T, p.E250Q, p.D246N, p.E256G, p.D266N)	15q24.3	CD2-BP1	Gain of function mutations of *CD2-BP1*, which interacts with pyrin and enhances pyrin inflammasome activation	Pyoderma gangrenosum, arthritis, acne	AD
**PASH**	*PSTPIP1* (p.A405C and increased monoallelic repetition of the CCTG microsatellite motif in the *PSTPIP1* promotor)	15q24.3	CD2-BP1	Deregulated *PSTPIP1* expression	Pyoderma gangrenosum, hidradenitis suppurativa, acne	AD
**PAPASH**	*PSTPIP1* (p.E277D)	15q24.3	CD2-BP1	Neutrophil activation by the Th17/TNF-α axis	Pyogenic arthritis, pyoderma gangrenosum, acne and hidradenitis suppurativa	AD
**PAMI**	*PSTPIP1* (p.E250K, p.E257K)	15q24.3	CD2-BP1	Deregulated *PSTPIP1* expression	Anemia, neutropenia, thrombocytopenia, high serum zinc, elevated calprotectin, arthritis, cutaneous inflammation, recurrent infection, failure to thrive, hepatosplenomegaly, lymphadenopathy	AD
**PAC**	*PSTPIP1* (p.G403R)	15q24.3	CD2-BP1	Deregulated *PSTPIP1* expression	Pyoderma gangrenosum, acne, ulcerative colitis	AD
**PFIT**	*WDR1*	4p16.1	WD40 repeat protein 1	Actin accumulation, pyrin inflammasome dysregulation, increased IL-18	Fever (up to 7 days), mucosal ulcerations, thrombocytopenia, infections	AR

AR, autosomal recessive; AD, autosomal dominant; ARPC1B, actin-related protein 2/3 complex subunit 1B; FMF, familial Mediterranean fever; PAAND, Pyrin-associated autoinflammation with neutrophilic dermatosis; MKD, mevalonate kinase deficiency; PK, porokeratosis; NOCARH, neonatal onset cytopenia with autoinflammation; cytopenia; rash; and hemophagocytes; PAPA, pyogenic arthritis; pyoderma gangrenosum and acne; CD2-BP1, CD2-binding protein 1; PASH, pyoderma gangrenosum; acne and hidradenitis suppurativa; PAPASH, pyogenic arthritis; pyoderma gangrenosum; acne; and hidradenitis suppurativa; PAMI, PSTPIP1-associated myeloid-related proteinaemia inflammatory syndrome; PAC, Pyoderma gangrenosum; acne; and ulcerative colitis; PFIT, periodic Fever Immunodeficiency and Thrombocytopenia; Th17, T helper 17 cells; TNF, tumor necrosis factor.

## 2 General principles of pyrin inflammasome activity

Innate immunity is essential to discriminating “self” from “nonself” antigens and can be considered a sophisticated system for sensing signals of danger, while remaining unresponsive to safe antigens and molecules. A series of germline-encoded pattern recognition receptors (PRRs) is engaged by the innate immune system to detect invariant microbial antigenic pattern (10). Such receptors include the membrane-bound Toll-like receptors (TLRs), which scan the extracellular setting for pathogen-associated molecular patterns (PAMPs), and some intracellular sensors, including the nucleotide-binding oligomerization domain (NOD)-like receptors (NLRs) and pyrin (10, 11). Thus, an important subgroup of intracellular PRRs are the NLRs, which recognize either PAMPs or danger-associated molecular patterns (DAMPs), expressed by the host cells when stressed (10). To neutralize pathogens, cells need molecular mechanisms that allow them to take decisive action and warn other cells quickly. Activating the inflammasome is one such way, with the assembly of a large multiprotein complex that provides a pivotal platform for the host innate immune system (5, 12–14). In general terms, inflammasomes detect threats via their protein sensor and promote inflammation, controlling the activation of the proteolytic enzyme pro-caspases and leading to a hypersecretion of proinflammatory cytokines (12, 15). The classical inflammasome complex consists of 3 major components:

• A variable cytosolic sensor (pyrin in the case of the pyrin inflammasome).
• The adaptor protein “apoptosis associated speck-like protein containing CARD” (ASC).
• The effector protein, mainly pro-caspase-1 (5, 14, 16, 17).

Inflammasomes often take their name from the sensor component, such as in the case of pyrin (12). The adaptor protein ASC is a bipartite molecule that binds pyrin via its N-terminal pyrin domain (PYD), and pro-caspase-1 via its C-terminal caspase activation and recruitment domain (CARD) (18, 19). However, before the inflammasome can assemble, it first needs to be primed. The priming step, occurring when pyrin senses PAMPs or DAMPs, transcriptionally upregulates the sensor components as well as proinflammatory cytokines production, mainly pro-interleukin (IL)-1β. The following step is sensing, which occurs when the sensor component senses various additional signals, as discussed below. The activation and regulation of pyrin present a multifaceted matter, wherein multiple molecules are capable of interacting with its diverse domains. Once the pyrin inflammasome is triggered, many copies of the sensor gather and each of them binds to ASC and to pro-caspases, assembling the multimolecular pyrin inflammasome that promotes the cleavage and thus the activation of pro-caspase-1 (14, 20). Inflammatory caspases are zymogens and must be cleaved to be activated: activated pro-caspase-1 promotes autocatalytic cleavage and “evolves” in its active form, caspase-1 (12, 19). Thus, caspase-1 cleaves pro-IL-1β and pro-IL-18 in their mature forms IL-1β and IL-18, stunning stimulators of inflammation (12, 13).

**Figure 1:**
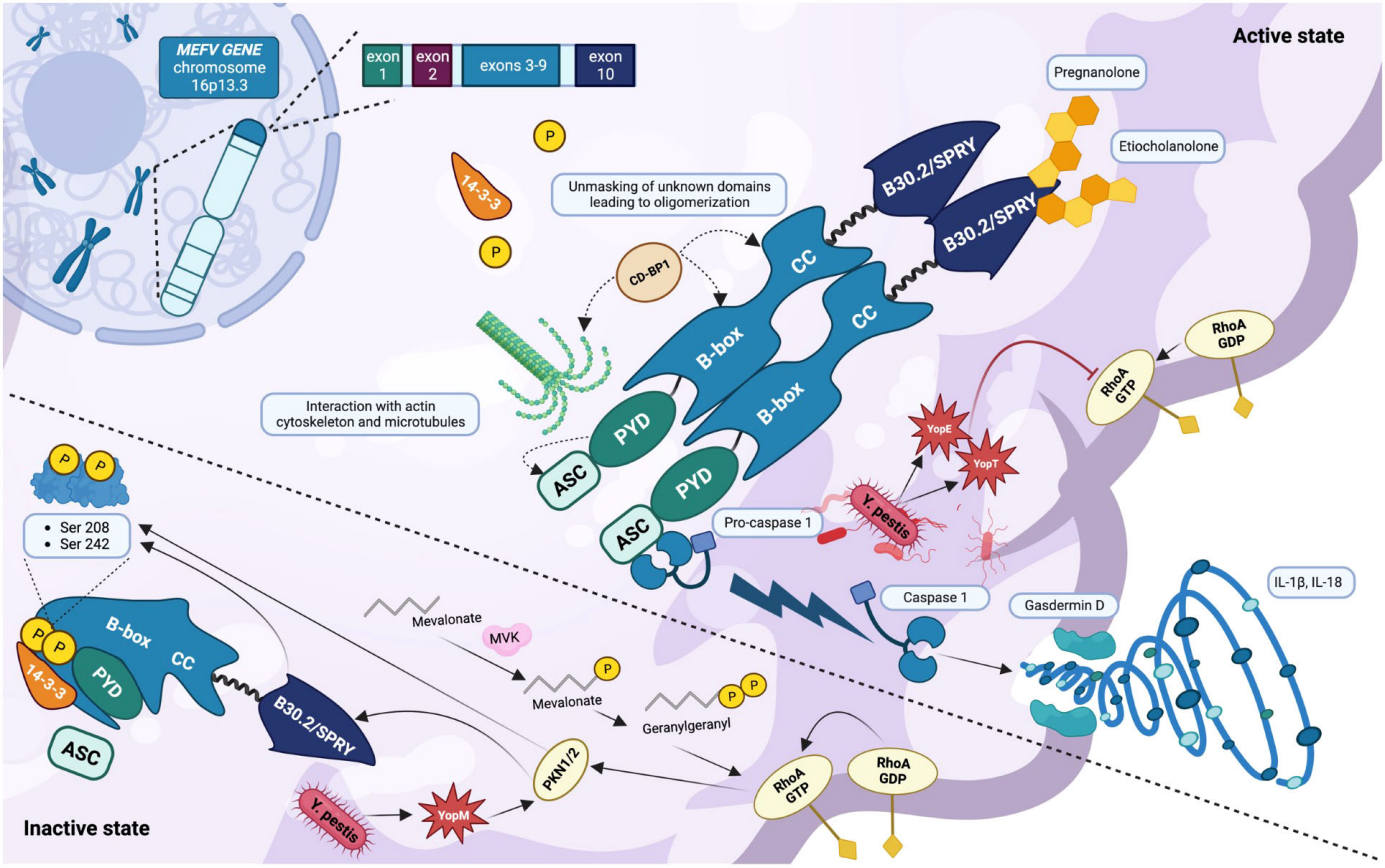
Inactive and active states of the pyrin inflammasome (created with BioRender.com). The *MEFV* gene is located on chromosome 16p13.3 and encodes the inflammasome sensor protein pyrin. The pyrin inflammasome is kept in an inactive state by the binding of the chaperone 14-3-3 to the phosphorylated residues Ser208 and Ser242. This phosphorylation is provided by the kinases PKN1/2, upon the stimulation of RhoA GTPases and the autoregulation of the B30.2/SPRY domain. In addition, the key domain PYD is covered by B-box. Pyrin is normally activated after bacterial inhibition of RhoA GTPase, resulting in a dephosphorylated protein that interacts with the cytoskeleton mainly via the CD-BP1 protein. The activation of the pyrin inflammasome results from its oligomerization and is responsible for the binding of its PYD domain to the PYD domain of the adaptor protein ASC. The final step consists of the cleavage and activation of pro-caspase-1 which in its active form is responsible for gasdermin D activation, cell membrane pores formation, and the activation and release of the cytokines IL-1β and IL-18. The high presence of health carriers of *MEFV* pathogenic variants around the Mediterranean Sea could be explained by a certain resistance to *Yersinia pestis*, which interacts with pyrin through its toxins YopE, YopT (inhibiting RhoA GTPases), and YopM (recruiting PKN1/2 and thus inhibiting pyrin). In the presence of various pathogenic *MEFV* variants, the steroid catabolites pregnanolone and etiocholanolone are able to activate pyrin through the B30.2/SPRY domain without involving RhoA GTPases.

IL-1β is considered one of the most powerful proinflammatory cytokine, activating the nuclear transcription factor NF-kB through the protein MyD88, inducing the production of several additional pro-inflammatory molecules, such as IL-6 and tumor necrosis factor (TNF)-α (12, 18). Otherwise, IL-18 induces vascular effects of inflammation, such as vasodilatation, increased expression of cells adhesion proteins and production of chemokines. IL-18 also induces interferon-gamma production, an important cytokine for dealing with viral infections (21). Caspase-1 also cleaves and actives gasdermin D (GSDMD), a protein that moves to the cell membrane and forms pores, causing an inflammatory form of programmed cell death called “pyroptosis”, an effective way to kill intracellular pathogens, and recruit other immune cells to the site of damage (**Figure 1**) (12, 14, 19, 22, 23).

Current research is giving more importance to these pathways, and a better understanding of all signals leading cells to undergo pyroptosis (19). It has been suggested that both IL-1β and IL-18 are released through the pores of gasdermin D (12, 19, 22). In addition to the classical inflammasome activation pathway, other additional caspases, including caspases-4 and -5 contribute to the inflammasome-dependent control of pyroptosis. Indeed, their interaction leads to self-cleavage of gasdermin D and promote pyroptosis (12, 24, 25). There are several different molecules which play a key role in pyrin’s regulation. They can be divided into negative regulatory pathways and positive regulatory pathways:

The main mechanisms which normally maintain pyrin inflammasome inhibited are retained the following:

• Pyrin is kept in an inactive state by the motif 14-3-3, which is bound to the phosphorylated pyrin.
• The phosphorylation of pyrin is provided in the residues S208 and S242 by the serine-threonine kinases PKN1 and PKN2.
• PKN1/2 are in turn kept in an active state by the phosphorylation of the RhoA GTPase (26, 27). Pyrin is strictly inhibited by RhoA GTPase and responds to alterations caused by infections and other cytoplasmatic disturbances. These processes, termed “homeostasis-altering molecular processes” (HAMPs) in the context of pyrin, lead to the inactivation of RhoA GTPase and reduce the stimulation/phosphorylation of the kinases PKN1/2 (27).

The main mechanisms which normally active pyrin and increase its activity are considered the following:

• Some pathogens and toxins interact and inhibit RhoA GTPase, thereby interfering with pyrin-mediated innate immune responses, via GTP hydrolysis and/or glycosylation of its Switch-I region.
• Cytoskeleton exerts a significant regulatory effect on pyrin: a proper microtubule network is required for pyrin activation, providing an activation signal to dephosphorylated pyrin so that shifting to an open conformation can bind ASC.

## 3 The *MEFV* gene and pyrin: critical loci, unique components

The *MEFV* gene, composed by 10 exons and located on chromosome 16p13.3, encodes the inflammasome sensor protein pyrin. Each of its exons is strictly related to a specific component of the protein and involved in different mechanisms and pathways (14, 27–29). Pyrin is a 781 amino acid protein mostly localized in the cytosol of various immune cells such as monocytes and neutrophils, in an inactive form (14). The protein was first identified in 1997 by The International FMF Consortium as a member of a family of nuclear factors homologous to the Ro52 autoantigen, almost exclusively expressed in granulocytes (7, 30). It is known that pyrin is composed by at least four main functional domains (27):

1. The N-terminal pyrin domain (PYD), amino acids 1-92.
2. The zinc finger domain (B-box), amino acids 370-412.
3. The α-helical coiled-coil domain (CC), amino acids 420-440.
4. The C-terminal domain (B30.2/SPRY), amino acids 597-776.

The purpose of this chapter is to analyze each of the *MEFV* regions*¸* their protein products, roles, and interactions.

### 3.1 The PYD domain: a bridge to ASC for pro-caspase-1 activation (1–92)

PYD is the N-terminal domain of pyrin which binds the homonymous PYD domain of ASC, also interacting with both the actin cytoskeleton and microtubules, although a nuclear translocation and interaction with nuclear transcription factors have been assumed (14). Interestingly, its original name was PAAD due to the protein families from which it was observed: P – pyrin, A – AIM, A – ASC, D – Death-domain like (27). Thus, ASC (via its PYD domain) binds PYD (pyrin) and oligomerizes into filaments, recruiting pro-caspase-1 via its CARD region (CAspase Recruitment Domain) (27, 31). PYD is fully encoded by *MEFV* exon 1 and only a small group of likely pathogenic/pathogenic variants have been reported in literature [Infevers - Tabular list (umai-montpellier.fr)], probably with a mechanism of abnormal binding to ASC. Nonetheless, this point has not yet been clarified.

### 3.2 Region of interest for 14-3-3 binding (93-369): S208 and S242 phosphorylation residues

Despite the fact that the pyrin region between amino acids 93-369 does not belong to any particular pyrin domain, its presence is essential for protein function. Indeed, two specific residues, Ser208 and Ser242, encoded by *MEFV* exon 2 serve as phosphorylation sites for PKN1/2. Once pyrin is phosphorylated, the chaperones 14-3-3 are able to bind and maintain its inactive state (14, 28, 29). Thus, the binding between phosphorylated pyrin and 14-3-3 is directly dependent on PKN1/2, which are stimulated and phosphorylated by RhoA GTPase. Interestingly, the binding between 14-3-3 and pyrin occurs on the corresponding murine amino acids, as demonstrated by recent murine studies (32). Furthermore, as discussed below, specific missense mutations S242R and E244K, involving this region of interest, cause the AID named “pyrin-associated autoinflammation with neutrophilic dermatosis” (PAAND) (33). In the meanwhile, the homozygous pathogenic variant S208T has been observed in children with a different clinical phenotype characterized by arthralgia, lymphadenopathy, purpuric rash, and infiltrates in bone marrow (14, 34, 35). It has been suggested that the S208 amino acid is not so central for pyrin inactivation than S242 (14). However, both patients with PAAND and homozygous S208T variants show increased IL-1β and IL-18 serum levels (14, 34–36). In summary, pathogenic variants in the region of interest between PYD and B-box prevent PKN1/2 from phosphorylating pyrin, preventing the binding of 14-3-3 and leading to pyrin hyperactivation.

### 3.3 The central region (370-596): B-box, CC, and the link with *PSTPIP1*/CD-BP1

The central region of pyrin (370-596) contains the B-box and CC domains (370-412 and 420-440, respectively). This portion of pyrin is essential for the protein’s correct oligomerization via ASC. In fact, the B-box domain is thought to play an autoinhibitory role by enveloping the PYD domain and thereby interfering with its binding to ASC. In addition, via B-box and CC, pyrin interacts with the regulatory protein CD2-binding protein 1 (CD-BP1), which is encoded by the *PSTPIP1* gene and primarily involved in the *PSTPIP1*-associated inflammatory diseases (PAIDs) (14, 35, 37). CD-BP1 and pyrin co-localize with the tubulin cytoskeleton and CD-BP1 triggers pyrin by binding B-box and CC, and therefore unmasking other domains, leading to its oligomerization (14, 38). Although a direct binding of CD-BP1 to B30.2/SPRY has been ruled out, the exact molecular mechanism of functioning has not been totally elucidated (28, 37). *PSTPIP1* is thought to co-organize the cytoskeletal structure in macrophages, and the cytoskeleton itself has been proposed as a critical factor in these pathogenic mechanisms (14, 39). As discussed below in detail, *PSTPIP1* pathogenic variants result in a hyperphosphorylated CD-BP1 and, therefore, a massive binding to B-box (29, 40). Heterozygous pathogenic variants of this key region P373L, H478T, and various T577 variants) have been associated severe presentations due to hyperinflammation with sterile serositis, colchicine resistance, and renal amyloidosis (28, 41–43). Interestingly, pathogenic phenotypes due to missense variants in this region are inherited in a dominant manner.

### 3.4 B30.2/SPRY (597-776) and the relevance of the mutations of exon 10

The C-terminal domain B30.2/SPRY (597-776) is encoded by the exon 10 and represents the hotspot for classical recessive-inherited *MEFV* pathogenic variants (28). Its function is still unclear, but there are much evidence that it represents a very important domain in the pathogenesis of diverse pyrin-related AIDs. Pathogenic variants of B30.2/SPRY induce a pathogen-independent activation of the pyrin responsible for FMF, and a direct binding with caspase-1 has been demonstrated *in vitro* despite the fact that the underlying molecular mechanisms are not fully understood (14, 27, 44–47). Recent research has attempted to elucidate the precise function of B30.2/SPRY fusing a human wild-type domain to murine pyrin, which naturally lack a B30.2/SPRY orthologous domain. The binding of PKN1 to pyrin was strongly reduced than in wildtype mouse pyrin, and it has been suggested that B30.2/SPRY could regulate the phosphorylation of the entire protein, and specifically that of the PKN1/2-binding residues (26). In addition, this study evaluated the phosphorylation levels of hybrid wildtype mouse pyrin with human B30.2/SPRY containing common pathogenic variants observed in FMF. Also in this instance, a decreased binding of PKN1/2 and, consequently, a disrupted binding of 14-3-3 to pyrin were observed (26). However, the exact mechanism that allows this key domain to regulate pyrin activity is not completely clear, and only some mechanisms have been hypothesized (14):

- B30.2/SPRY could act as a platform allowing the binding of PKN1/2 and 14-3-3 to pyrin, regulating their affinity to the protein.
- After the binding between 14-3-3 to S208 and S242, B30.2/SPRY could interact with B-box and CC, or other regions, to organize a secondary protein structure that autoinhibits pyrin. This mechanism has been also suggested for two other NLRP1-related AIDs (48).

Nevertheless, it is currently unknown if B30.2/SPRY acts with a proinflammatory or missed autoinflammatory mechanism (49). A further investigation has elucidated an atypical method of pyrin activation, suggesting that endogenous steroid catabolites have the potential to induce autoinflammation via the pyrin inflammasome. This finding provides an explanation for the phenomenon known as “steroid fever,” which was first reported in the late 1950s following the administration of steroids to human subjects (50). Interestingly, high levels of pregnanolone and etiocholanolone, two catabolites of progesterone and testosterone, have been documented to active pyrin through the B30.2/SPRY domain, without involving RhoA GTPases, in PAAND and FMF patients (50).

### 3.5 RhoA GTPase regulation and *Yersinia Pestis*: why so many FMF health carriers?

RhoA GTPase is the target of several pathogens that aim to impair multiple defense mechanisms of the infected cell. The effects of such pathogens result in an impaired activity of this molecule, thus reducing its continuous stimulation of PKN1/2 and activating the pyrin inflammasome. This is an ancient mechanism of human organism to respond to pathogens and their toxins, and it could explain why up to 10% of the population of some countries has heterozygous pathogenic *MEFV* variants, typically observed in FMF (51, 52). This mechanism allowed individuals to have a greater immune response to some pathogens, in the case of *MEFV* mutations it was *Yersinia Pestis*. The main toxins interacting with RhoA GTPase are discussed below:

- *Yersinia Pestis* promotes Rho GTPase impairment via two distinct toxins: YopE is responsible for GTP hydrolysis, whereas YopT catalyzes cleavage and disrupts the localization of RhoA from the cell membrane. *Yersinia Pestis* secretes a third toxin called YopM, which inhibits caspase-1 and increases pyrin phosphorylation by recruiting and interacting with PKN1/2. Gain-of-function B30.2/SPRY pathogenic variants circumvent YopM inhibition, resulting in *Yersinia Pestis* infection resistance (14, 51, 53). This is not the case for E148Q carriers.
- *Clostridium difficile* expresses the virulence factors TcdB, which promotes GTP glycosylation and, consequently, its inactivation.
- Other bacterial molecules interact with the Switch I region of RhoA GTPase, reducing its activity by unclear mechanisms (such as *Bordetella pertussis, Clostridium botulinum*, and others).

In addition, RhoA GTPase is dependent on a variety of enzymatic regulatory mechanisms. Rho GTPase requires geranylgeranylation for precise translocation to the cell membrane and appropriate function. This enzymatic process is in turn dependent on the enzyme geranylgeranyl pyrophosphatase, which is involved in the formation of cholesterol and is significantly reduced in MKD patients due to impaired functioning of the *MVK* gene (54). Growing evidence suggests that RhoA GTPase does not interact directly with pyrin, which is dependent on HAMPs in a particular immunity context.

## 4 The importance of cytoskeleton and actin filaments

**Figure 2:**
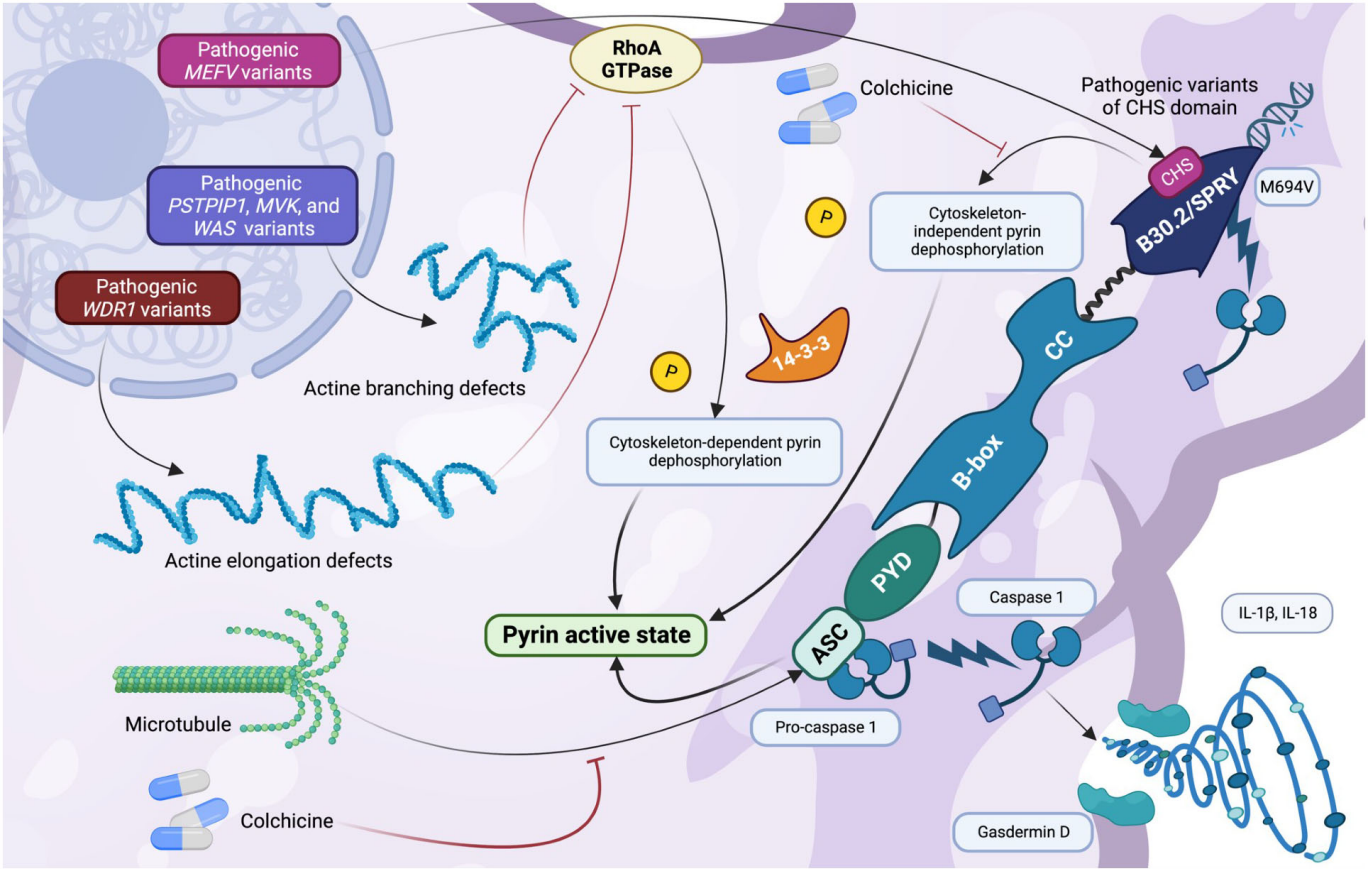
The role of the cytoskeleton in pyrin regulation (created with BioRender.com). Cytoskeleton exerts a significant regulatory effect on pyrin: a correct microtubule network is required for pyrin activation, providing an activation signal to dephosphorylated pyrin so that shifting to an open conformation can bind ASC. A disruption in the multiple genes involved in pyrin regulation can impair the cytoskeletal regulatory pathways. Multiple pathogenic *MEFV* variants related to B30.2/SPRY (observed in familial Mediterranean fever) and to the recently described CHS domain result in cytoskeleton-independent pyrin dephosphorylation. In contrast, a disruption in the *WDR1* gene results in actine elongation defects, whereas that observed in the *PSTPIP1*, *MVK*, and *WAS* genes causes actine branching defects. Actine disruption leads to ineffective RhoA GTPase regulation and inhibition, leading to cytoskeleton-dependent pyrin dephosphorylation and activation. Colchicine disassembles the microtubule network and actin regulation of pyrin through unfathomable mechanisms.

The cytoskeleton plays a crucial role in numerous cellular activities that occur during a leukocyte response, including migration, extravasation, and phagocytosis, and it has been described that actin remodelling defects play an important role in the pathogenesis of several AIDs (27, 55). A growing number of immunity disorders, presently referred to as actinopathies, have been associated with abnormalities in the functions of the actin cytoskeleton (56, 57). Specifically, there is growing interest in understanding how a dysregulation of cytoskeleton is linked to an hyperactivation of pyrin in patients with impaired functioning of *MEFV, PSTPIP1*, and *MVK* (55). It is widely accepted that pyrin does not directly binds RhoA GTPase but rather recognizes its impairment, also resulting in an actin cytoskeleton pathway disruption (58). Rho GTPases have been advocated as regulatory molecules of cytoskeleton organization, primarily in NOD1-focused experimental studies (27, 59). This distinctive inflammasome sensor protein detects intracellular pathogen effectors by monitoring Rho GTPase activity. NOD1 has been shown to form a multiprotein complex with Rho GTPases that is sensitive to cytosol modifications resulting from pathogen manipulation of the actin cytoskeleton (59). In the meantime, it has been proposed that the NLRP3 inflammasome may depend on microtubule organization (60). Due to its hypothetical interactions with cytoskeleton and RhoA GTPase, pyrin could be regulated in a comparable manner, with microtubule control dependent on its dephosphorylation in response to RhoA GTPase inactivation and HAMPs detection. It’s important to remind that leukocyte response to pathogen-related pyrin activation in health individuals, such as that of Clostridium, necessitates the proper functioning of intact cytoskeletal microtubules network (61). In contrast, it has been demonstrated that in patients with FMF-related pathogenic variants in the B30.2/SPRY domain, pyrin activation does not require functional microtubules for a proper activation (61). In fact, whereas microtubule assembly inhibitors such as colchicine normally prevent pyrin-mediated cleavage and activation of pro-caspase-1, microtubule disassembly does not inhibit or depend on the inflammatory cascade in FMF (61). This is a peculiar characteristic observed in FMF patients but not in those with other AIDs, most likely due to mutation sites in the key B30.2/SPRY domain (61). The pathogenic mechanisms involve pyrin dephosphorylation, which is normally controlled by microtubules. However, in FMF, pyrin dephosphorylation is independent of the cytoskeleton, resulting in microtubule-independent nucleation of ASC specks and inflammasome signaling (61). In summary, microtubules transmit an activating signal to dephosphorylated pyrin (which self-inhibits) so that it adopts an open conformation. For unknown reasons, FMF pathogenic variants affecting the B30.2/SPRY domain cause dephosphorylated pyrin to remain in an open conformation, resulting in microtubule-independent binding to ASC and inflammasome activation (61, 62). Despite the fact that colchicine should not work in FMF based on these observations, the molecular mechanism behind its effect is unknown. Following studies have evaluated how *MEFV* pathogenic variants do not result in constitutive pyrin activation, but rather a decrease in the activation threshold via different mechanisms, supporting these observations (5, 49). Recently, a negative regulatory function has been suggested for B30.2/SPRY together with the description of a novel central scaffold domain (CHS) of pyrin. According to this research, five pathogenic variants of CHS cause uncontrolled pyrin activation only depending on its dephosphorylation (5). However, this observation should still be confirmed by additional research. Relevant is the stupefying effect of colchicine on inflammation caused by pyrin. Colchicine is the drug of choice for patients with FMF, but its precise mechanism of action has not yet been elucidated, and patients with MKD and other pyrin-related AIDs have poor responses to this medication. Microtubules are disrupted by colchicine, and it has been demonstrated in murine models that microtubule dynamics may regulate pyrin activation via actin disassembly (61, 63). Consequently, it can be inferred that colchicine operates in a subsequent stage, potentially influencing either the pyrin oligomerization or ASC recruitment processes. Alternately, colchicine may promote the dephosphorylation of pyrin, allowing its transport to a subcellular location that facilitates activation, similar to the mechanism observed in NLRP3 (60, 64). Growing knowledge of the pathways underlying the periodic fever, immunodeficiency, and thrombocytopenia (PFIT) syndrome provides additional evidence for the influence of the cytoskeleton on pyrin regulation (55, 65, 66). This disease was previously known as “lazy leukocyte syndrome” and is a rare inherited immunologic disorder caused by mutations in the WD repeat-containing protein 1 (*WDR1*) gene on chromosome 4p16.1. This gene encodes WDR1, a protein implicated in cofilin-mediated F-actin depolymerization, which has been linked to pyrin inflammasome regulation through a mechanism that is not entirely clear (66). Affected monocytes and lymphocytes frequently exhibit increased podosome volumes and polymerized F-actin levels, resulting in aberrant intracellular WDR1 aggregates and elevated pyrin inflammasome activity (65). Inadequate phagocytosis and lysozyme synthesis have also been linked to decreased neutrophil chemotaxis and a slight decrease in neutrophil bactericidal activity (65, 67). Interestingly, the *WAS* gene, which encodes the WASP protein and is activated by *PSTPIP1*, may play a vital role in these mechanisms (55). Pathogenic variants occurring in this gene are related to a disrupted WASP protein, expressed in hematopoietic cells, and related to X-linked thrombocytopenia, X-linked neutropenia, and Wiskott-Aldrich syndrome, which is characterized by immunodeficiency, eczema, and thrombocytopenia (55, 68). In this disease, loss-of-function pathogenic variants of *WAS* are responsible for cytoskeletal defects of megakaryocytes (and thus thrombocytopenia), decrease in T-cell migration, B and T cell leukopenia, defective NK cells, and neutropenia (55, 68, 69). However, while *WDR1* is involved in actin elongation defects, *MEFV*, *PSTPIP1*, *MVK*, and *WAS* have been advocated for actin branching defects (**Figure 2**) (55). An additional crucial protein for branching of F-actin is encoded by the “actin-related protein 2/3 complex subunit 1B” gene (*ARPC1B*) and serves as a component of the actin-related protein 2 (ARP2)-APR3 complex. The few patients described with pathogenic variants of *ARPC1B* manifest with eosinophilia, platelet abnormalities due to impaired spreading function, systemic autoinflammation, lymphoproliferation, and immune deficiency (2, 55, 70–72). A recently described AID, named “neonatal onset cytopenia with autoinflammation, cytopenia, rash, and hemophagocytes” (NOCARH) has been related to pathogenic variants of the C*DC42* gene and are likely linked to actin dysregulation. In such patients, high levels of IL-18 have been documented, with a complete resolution after treatment with the IL-1 inhibitors anakinra and canakinumab (73). Given that *CDC42* encodes a small Rho GTPase, named cell division control protein 42 homolog (CDC42), governing actin polarization, cytoskeletal architecture, and cell division, the mechanism of this type of inflammation might be related to the abnormal activation of the pyrin inflammasome, with unknown mechanisms (2, 73–75). Other studies emphasize the importance of the centrosome, the most important microtubule organizing center, in regulating pyrin (76, 77). Indeed, centrosomes may serve as platforms for the final assembly and activation of the pyrin inflammasome. This evidence arises from the fact that ASC, caspase-1 and IL-1β colocalizes with the centrosome in a specific cell population. This finding may explain in part how colchicine, a potent microtubule-disrupting drug, inhibits pyrin activation (76).

## 5 Pyroptosis, gasdermin D and alarmins,which role in pyrin activation?

**Figure 3:**
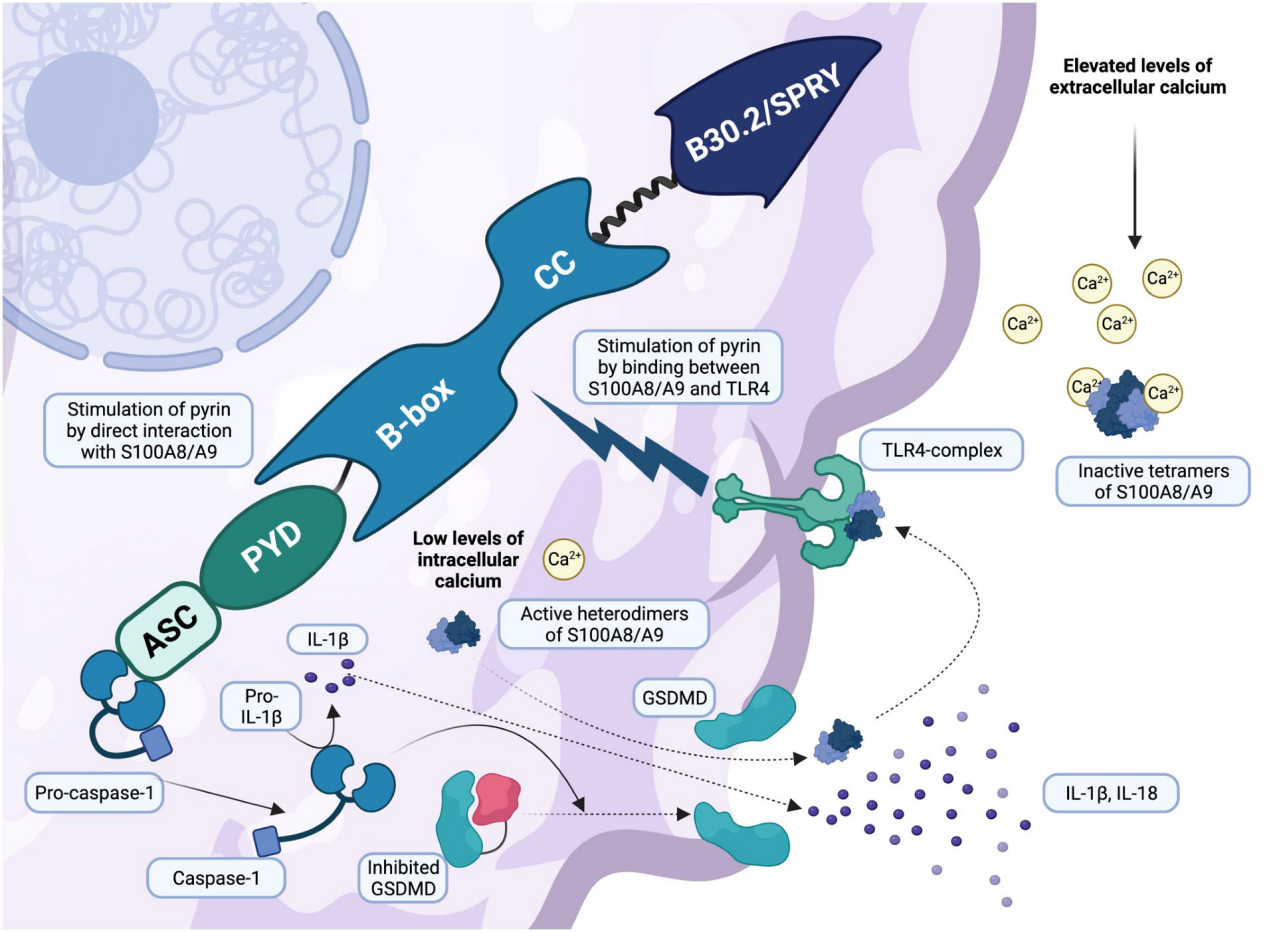
Active gasdermin D is responsible for pyroptosis and alarmins S100A8/A9 secretion (created with BioRender.com). Once active, pro-caspase-1 cleaves gasdermin D (GSDMD), leading to its activation. GSDMD is responsible for cell membrane pores and a peculiar inflammatory pathway called pyroptosis. Leukocytes secrete a variety of pro-inflammatory cytokines, primarily IL-1β and IL-18, through the membrane pores, increasing systemic inflammation. However, recent research has shown that alarmins S100A8/A9 are secreted in a similar manner. Low levels of intracellular calcium are responsible for the assembly of active heterodimers S100A8/A9, which leave the cell via the GSDMD pores and bind the TLR4 receptor, triggering an additional inflammatory response and pyrin stimulation. In contrast, elevated levels of extracellular calcium lead to the formation of inactive tetramers (S100A8/A9)_2_, which act with negative regulatory mechanisms on S100A8/A9 alarmins.

Pyroptosis is an intriguing phenomenon that develops from complex interactions involving GSDMD and the formation of cell membrane pores. The discovery of GSDMD functioning has largely clarified the final part of the pyrin activation cascade: in particular, GSDMD is required for the release of IL-1β and the alarmins S100A8/A9 and S100A12 outside the cell via the formation of several cell membrane pores, upon the cleavage and stimulation of caspase-1 (23). This evidence could result in the development of GSDMD blockers that could be effective against pyrin-related hyperinflammation (23). Interestingly, during autoinflammatory flares FMF patients share exorbitant high levels of the alarmins S100A8/A9, even higher than controls but also than patients with other AIDs and sepsis (22, 78). Patients with systemic onset juvenile idiopathic arthritis and Still’s disease are estimated to have up to 20-fold lower blood levels of S100A8/A9 than FMF, whereas patients with sepsis or cryopyrinopathies have up to 200-fold lower blood levels (79–81). When in an active state (concomitating with low intracellular calcium levels), the mechanism of action of S100A8/A9 involves the formation of intracellular heterodimers. These heterodimers are released by monocytes and neutrophils and have the ability to bind and activate TLR4 as well as other interconnected inflammatory pathways. Elevated levels of extracellular calcium result in the formation of inactive tetramers, constituting the negative regulation mechanism. These tetramers inhibit their binding to TLR4, thereby reducing their proinflammatory effects (22, 80, 82, 83). S100A8/A9 have two regulatory calcium-binding sites and are involved in multiple molecular pathways, including cytoskeletal-membrane interactions (84). In recent years, their secretion mechanism remained unknown despite the exclusion of the classical endoplasmic reticulum/Golgi route due to the absence of a specific sequence (84). Recent research has shown that pyrin interacts directly with heterodimers S100A8/A9 and promotes their secretion through GSDMD membrane pores (**Figure 3**) (22). This study provided a great deal of novel information regarding this intricate argument:

- S100A8/A9 heterodimers directly interact with pyrin in a calcium-dependent manner.
- Pyrin assumes a pivotal function in the extracellular release of S100A8/A9 via the GSDMD pores.
- Pyrin dysregulation observed in FMF results in a hypersecretion of S100A8/A9 and abrogates their negative regulatory mechanisms.
- Caspase-1, which is responsible for GSDMD cleavage and activation, could be involved in the secretion process of S100A8/A9, with an intriguing link to FMF molecular background.
- Colchicine reduces the expression of S100A8/A9 in leukocytes in healthy controls but not in those with FMF.
- In stimulated leukocytes, pyrin and S100A8/A9 reside in the same protein complex associated with cytoskeletal tubulin-forming microtubules.
- The inflammatory phenotype observed in FMF-related pyrin hyperactivation is strongly increased in the absence of regulatory tetramers, not related only to the enhancement of IL-1β expression.
- The absence of functioning GSDMD in murine models with homozygous pathogenic FMF variants reverses the inflammation processes induced by pyrin abnormal activation.
- Other AIDs underscoring an abnormal action of caspase-1 and GSDMD do not share significantly elevated S100A8/A9 serum levels, implying that there may be additional pyrin-specific mechanisms involved.
- FMF-related pathogenic variants result in the elimination of the reliance of S100A8/A9 secretion on a functional microtubule system.

Moreover, the serum levels of alarmin S100A12 have been reported to be higher in FMF patients than in healthy controls (85). Inflammasomes are a fascinating phenomenon, whose disruption result in a variety of clinical phenotypes that reflect their distribution and the severity of associated genetic defects.

## 6 Pyrin-related inflammasomopathies

### 6.1 Familiar Mediterranean fever

FMF is the most known monogenic AID, caused by gain of function mutations of the *MEFV* gene, mostly affecting exon 10 and thus the B30.2/SPRY domain (14, 44, 86). This disease is quite common among populations surrounding the Mediterranean Sea. Indeed, in Turkey there is a very high prevalence between 1:400 and 1:1000, with similar prevalence rates reported in Israel and Armenia (86, 87). As described above, the high carrier frequency for *MEFV* mutations among Ashkenazi Jews and Turks may be explained with a selective advantage against *Yersinia pestis*, able to neutralize the classical pyrin inflammasome assembly producing a set of virulence proteins that interact with RhoA GTPases. FMF clinical phenotype often appears in pediatric age and is characterized by recurrent inflammatory attacks manifesting with high-grade fever, polyserositis, arthritis, and erysipelas like erythema (ELE) (88). High-grade fever and abdominal pain, due to sterile peritonitis, are by far the most common manifestations, reported in more than 90% of patients (86). Notably, a history of abdominal surgery is common among FMF patients (44). Comorbidities may occur (89). FMF clinical onset usually occurs before 5 years of age, and up to 90% of patients become symptomatic before the age of 20 years (88). The most feared complications of this disorder are represented by renal amyloid A amyloidosis and end-stage chronic renal disease, fortunately not common due to a growing number of correct diagnoses (44, 90, 91). Although FMF has an intermittent-recurrent inflammatory pattern, persistent inflammation between attacks can be detected in up to 30% of children, influencing growth and bone health, and evidencing a constant elevation of the indices of inflammation S100A8/A9 and serum amyloid A (SAA), with increased risk of developing renal amyloidosis over time (91, 92). The heterogeneous clinical expression is attributed to the various interactions between genetic and environmental factors, but the phenotype seems to be mainly determined by the *MEFV* variants carried (44). Indeed, among the 14 most common pathogenic variants observed in *MEFV*, 80% of them occurs in exon 10 and up to 20% in exons 2, 3 and 5 (93, 94). Many *MEFV* mutations have no certain pathogenic role, and the interpretation of genetic sequencing must be done very carefully (93, 94). M694V is a quite common and penetrant pathogenetic *MEFV* mutation that is associated with severe manifestations and early presentation (94). In addition, also biallelic exon 10 variants have been associated to severe course and early onset (44). The E148Q variant occurs in exon 2 and is one of the most common variants observed in *MEFV*, but it also occurs frequently in general population and its pathogenic role is still controversial (94). In a recent study, E148Q was not found to be related to FMF development, and E148Q/M694V patients were reported to be comparable to those with the M694V variant alone (95). The conclusions drawn from this study have generated controversy as a result of the small study population of E148Q/M694V patients (96). Further reports have related E148Q to FMF with a mild course, but it remains debatable whether the compound E148Q/M694V heterozygosity is associated with colchicine resistance (97–99). It is highly probable that the genotype alone is not sufficient for determining the entire clinical phenotype; thus, a multifaceted interplay exists among genetic background, environmental factors, and other modifier genes. Notably, patients from Turkey and Armenia have been more frequently reported to share FMF phenotypes with E148Q variants compared to patients from other countries (96, 100).

FMF classification criteria have changed over time: historical criteria did not consider the patient’s genotype, nowadays essential for an accurate diagnosis (9). In 2019, Gattorno et al. developed and validated the new Euro-Fever/PRINTO (Paediatric Rheumatology International Trials Organization) evidence-based classification criteria for hereditary recurrent fever, combining an international expert consensus and statistical evaluation of real patients, including for the first time the clinical genotype assessment (9). Therefore, FMF can be currently diagnosed when a confirming *MEFV* genotype is present, with at least one of the following symptoms: episodes lasting 1-3 days, arthritis, chest pain, or abdominal pain. Nevertheless, two of these requirements must be present when no confirmed *MEFV* genotype is found (9). A second set of criteria based only on clinical criteria was provided as a possible aid for the indication for molecular analysis or when genetic testing is not easily accessible (**Table 2**) (9). In addition, recent evidence admitted an autosomal dominant (AD) pattern of transmission in presence of gain-of-function mutations of *MEFV* with a dose-dependent effect (9, 47, 101).

**Table 2:** Euro-Fever/PRINTO Classification criteria for familial Mediterranean fever (9).

EuroFever/PRINTO Classification criteria for familial Mediterranean fever	
Classification criteria	Clinical classification criteria* (at least six out of nine)
Presence of confirmatory *MEFV* genotype and at least one among the following: ◼ Duration of episodes 1–3 days ◼ Arthritis ◼ Chest pain ◼ Abdominal pain *OR* Presence of not confirmatory *MEFV* genotype and at least two among the following: ◼ Duration of episodes 1–3 days ◼ Arthritis ◼ Chest pain ◼ Abdominal pain	Presence: ◼ Duration of episodes 1–3 days ◼ Arthritis ◼ Chest pain ◼ Abdominal pain ◼ Eastern Mediterranean ethnicity Absence: ◼ Maculopapular rash ◼ Urticarial rash ◼ Aphthous stomatitis ◼ Painful lymph nodes

*Clinical classification criteria have less sensitivity, specificity, and accuracy and should be used to guide molecular analysis testing or if no genetic analysis is available.

EULAR endorsed recommendations for the management of FMF has been published (102). Helpful recommendations for a better comprehension of *MEFV* mutations have been published (Infevers - Tabular list (umai-montpellier.fr)) (94). Given that even heterozygous genotypes have an increased risk of AA amyloidosis, treatment should be administered also in paucisymptomatic patients to prevent complications (91, 94). Colchicine is the first line of treatment for FMF and should be started as soon as possible and continued indefinitely (44, 91). In fact, its efficacy and safety have long been established, with reduction in the risk of renal amyloidosis to less than 1%, but no agreement over time to discontinue colchicine, with most of patients subjected to lifelong prophylaxis (44, 47, 91, 103, 104). As discussed above, colchicine inhibits neutrophil chemotaxis and extravasation, decreasing their flexibility through tubulin breakdown and microtubule instability, inhibiting pyrin inflammasome hyperactivation. Diarrhoea (up to 10,8%) and increased transaminases (up to 6%) are the most frequent side effects, although also important toxicities have been rarely observed (104–106). Particular attention should be paid to interaction with other drugs or foods such as macrolides, cyclosporin, statins, omeprazole, and even grapefruit. Moreover, almost 20% of patients are unable to maintain therapy, usually due to persistent diarrhoea (44, 103). In clinical practice, the dose of colchicine can be divided between two and three times daily to reduce side effects. However, about 5%–10% of patients do not respond to colchicine and need different therapeutic approaches (103, 107). M694V homozygous patients, biallelic variants of exon 10, and severe disease courses are at higher risk to be colchicine nonresponsive, while patients with serositis and heterozygous variants are usually more sensitive to lower drug doses (103, 107). Colchicine-resistant (crFMF) and intolerant patients are treated with biologic drugs, mostly IL-1 inhibitors (103). Several studies have been published on safety and efficacy of anakinra, canakinumab and rilonacept in crFMF, with good results (6, 103, 108–111). IL-1 inhibitors improve renal involvement and quickly resolve acute attacks, with low reported rates of collateral infections (111–113). The efficacy of on-demand use of anakinra has been documented in a cohort of crFMF patients, with a significantly long-term improvement (114). Interestingly, a single-dose anakinra has been recently retrospectively evaluated, resulting in a reduction in the duration and severity of inflammatory attacks (115). A double blind randomized trial evaluated the use of anakinra in crFMF, reporting good improvement and safety (116). Efficacy was also confirmed by following observational studies (117, 118). The “Canakinumab Pivotal Umbrella Study in Three Hereditary Periodic Fevers (CLUSTER)” was a lead randomized placebo-controlled study that evaluated the efficacy and safety of canakinumab in 63 crFMF patients. After 16 weeks of treatment with canakinumab at 150 mg/monthly, 61% of them reported a full response; while at 300 mg/monthly, 71% of patients was in full health (6). Thus, canakinumab has received approval by the “U.S. food and drug administration” (FDA) for crFMF (6), while both anakinra and canakinumab are currently approved for FMF in Europe by the “European medicines agency” (EMA) (110). A systematic review and meta-analysis were conducted in 2018 to assess the safety and efficacy of treatments available for FMF patients. Colchicine resulted in a reduction in the number of symptomatic patients independently of its administration strategy (single-dose or divided into three doses) (119). Nevertheless, only two studies using anakinra and rilonacept were included, not resulting in a significant reduction in the severity and frequency of FMF attacks (119). However, since 2018, many studies have demonstrated the efficacy of IL-1 inhibitors on FMF, as discussed above.

### 6.2 Pyrin-associated autoinflammation with neutrophilic dermatosis

Two distinct mutations in *MEFV* exon 2, S242R and E244K, have been linked to the development of PAAND (120). This is an AD inherited disease, characterized by early onset of periodic fever, severe acne, neutrophilic dermatosis, pyoderma gangrenosum (PG), non-infectious cutaneous abscesses, and arthromyalgia (120). Its pathogenetic mechanism consists in the loss of a 14-3-3 binding motifs at phosphorylated pyrin, with the consequent pyrin inflammasome activation and IL-1β hyperproduction (36). PAAND is usually treated with IL-1 inhibitors, even though oral retinoids and TNFα inhibitors may effectively control dermatosis (36, 120). Nonetheless, over 385 *MEFV* variants have been recorded, although the real role of the majority of them remains unclear (121).

### 6.3 Mevalonate kinase related disorders: mevalonate kinase deficiency and porokeratosis

MKD is a rare AR inherited AID, caused by loss of function mutations in the *MVK* gene, located on chromosome 12q24.11 and encoding the enzyme mevalonate kinase (MVK) (122). MVK is physiologically involved in the isoprenoid biosynthesis pathway, catalysing ATP-dependent phosphorylation of mevalonate to produce 5-phosphomevalonate, essential in cholesterol biosynthesis (123). Its deficiency reduces the prenylation of proteins such as geranylgeranyl pyrophosphatase, necessary for the activation of RhoA GTPase and thus the inhibition of the pyrin inflammasome (26, 123, 124). To date, more than 175 pathogenic or likely pathogenic *MVK* variants have been described: most are substitutions, and small intragenic rearrangements, including three deletions of exons 2, 3 or 5 (Infevers - Tabular list (umai-montpellier.fr) (122). The most frequent *MVK* variant V377I is derived from a founder effect (125). In addition, also I268T, H20P, H20N, and P167L have been reported in in up to 71% of MKD patients (122, 126–128). However, few studies of phenotype-genotype correlation are currently available and more accurate details are needed (122). Originally, two distinct syndromes have been identified, classic “mevalonic aciduria” (MA) and “hyperimmunoglobilinemia D with periodic fever syndrome” (HIDS), but after the discovery of a common cause, recognized in the deficiency of MVK, they have been respectively considered as the severe and mild form of the same disease (122). MKD is a spectrum of disorders, ranging from mild to severe phenotype, depending on residual enzyme activity (122). Autoinflammatory symptoms, such as recurrent high-grade fever lasting 3-7 days, arthritis, abdominal pain, hepatosplenomegaly, and lymphadenopathy, are prevalent in milder HIDS forms, with preserved enzyme function (usually greater than 1%) (122, 124, 126). When no residual enzymatic function is present (< 1%), the clinical phenotype is characterized by MA, with recurrent high-grade fever, arthritis, growth failure, dysmorphism and severe cognitive disability (122, 124, 126, 129). The onset of autoinflammatory attacks is usually within the first 6 months of life, and may relapse every 1-2 months (124, 126). Several triggers have been associated with the appearance of inflammatory attacks, such as viral infections, emotional stress, and surgery (122). For a correct diagnosis of MKD, both genetic and clinical criteria have been established by the new Euro-Fever classification for HRF (**Table 3**) (9). Renal AA amyloidosis is rarer than in FMF, although patients carrying the V377I and I268T variants are considered at higher risk (91, 127, 130). Proinflammatory cytokines, such as IL-1β, IL-6 and IFN γ, are dramatically increased during attacks, and a strong molecular connection has been attributed to FMF and MKD, basing on their pathophysiological mechanisms (123). Nevertheless, colchicine is not helpful in MKD patients (26, 123).

**Table 3:** Euro-Fever/PRINTO Classification criteria for mevalonate kinase deficiency (9).

EuroFever/PRINTO Classification criteria for mevalonate kinase deficiency	
Classification criteria	Clinical classification criteria* (at least six out of nine)
Presence of a confirmatory *MVK* genotype and at least one among the following: ◼ Gastrointestinal symptoms ◼ Cervical lymphadenitis ◼ Aphthous stomatitis	At least three out of six: ◼ Gastrointestinal symptoms ◼ Painful lymph nodes ◼ Aphthous stomatitis ◼ Age at onset < 1 years ◼ Triggers ◼ Maculopapular rash

MVK, mevalonate kinase.

*Clinical classification criteria have less sensitivity, specificity, and accuracy and should be used to guide molecular analysis testing or if no genetic analysis is available.

Dominant pathogenic *MVK* variants have been recently related to the development of PK, a severe localized skin disorder totally different from the systemic form MKD (122, 131). This rare dermatologic disorder is considered dominantly inherited but recessively expressed following a second acquired somatic mutation (128). Environmental factors, such as ultraviolet A (UVA)-mediated DNA alterations of epidermis cells, appear to play a key role in precipitating cell death in patients with heterozygous pathogenic germline *MVK* variants (122). Mevalonic acid may have a protecting role in keratinocytes cell damage induced by UV radiations (129). Keratotic lesions appear in adolescents as small atrophic plaques with a keratotic rim, mainly localized on sun-exposed areas, but new lesions may also appear in adult age (122). To date, several clinical trials have examined the use of IL-1 inhibitors in the treatment of MKD (132). An analysis of the effectiveness of various therapeutic strategies was conducted on a cohort of 103 patients from the International MKD/HIDS Registry, and those receiving conventional therapies showed little to no response, except for high doses of glucocorticoids and biologics (126). In addition to FMF, the CLUSTER study has also evaluated the efficacy of canakinumab in 72 MKD patients (6). After 16 weeks, substantially more patients receiving canakinumab at 150 mg/monthly had better responses than those receiving placebo, and more than 80% of responders continued to be responsive to canakinumab for up to 8 weeks (6). No serious opportunistic infections or fatalities have been reported, and canakinumab received FDA and EMA approval for the treatment of MKD. In addition, IL-1 inhibitors represent a hope for future local therapy for PK patients (122). Treatment for *MVK-*related diseases may have exciting prospects. A study was conducted on two sisters carrying the same homozygous genotype (V377I/V377I), showing opposite phenotypes: one sister was asymptomatic, while the other presented with a classical phenotype (133). Thus, a *STAT1* missense mutation R241Q was identified, influencing the phenotype of the symptomatic patient. Such mutation provided an hyperactivation of the Janus kinase/signal transducer and activator of transcription (JAK/STAT) signaling pathway, which has a central role in inflammation (133). Thus, JAK/STAT pathway may strongly influence MKD phenotype, with good therapeutic perspectives (133, 134).

### 6.4 *PSTPIP1*-associated autoinflammatory diseases

Gain of function mutations affecting the proline-serine-threonine phosphatase interacting protein 1 (*PSTPIP1)* gene are responsible for the “pyogenic arthritis, pyoderma gangrenosum and acne” (PAPA) syndrome, a rare AD inherited autoinflammatory disease. As discussed previously, *PSTPIP1/*CD2-BP1 is involved in cytoskeletal organization and acts has a positive regulatory function for pyrin inflammasome activation. As observed in pyrin-deficient mice, the enhanced interaction of *PSTPIP1* with pyrin may contribute to a PAPA clinical phenotype by sequestering pyrin and tipping the regulatory scales in favour of an increased IL-1β production by peripheral blood leukocytes (37). Joint and skin involvement are hallmarks of PAPA syndrome. The former usually appears in childhood as sterile oligo arthritis, before healing in adult age. Instead, skin manifestations may include oral lesions, pyoderma gangrenosum, aseptic abscesses, and severe cystic acne. Acne in PAPA syndrome occurs from puberty onwards, developing at about 13 years of age with often a nodulocystic phenotype, while skin ulcers typically develop during adolescence, frequently leaving unsightly scars and psychosocial involvement (135). Less than half of patients show moderate acne, while only a few cases of acne fulminans have been described in PAPA syndrome (136). The two main relevant pathogenic *PSTPIP1* variants increasing the binding of CD-BP1 to pyrin are considered A230T and E250Q (37, 81). In the meanwhile, many other heterozygous variants have been reported and identified as pathogenic, such as Y119C, E277D, and R228C (81, 135). PSTPIP1-associated autoinflammatory diseases (PAIDs) represent a wide group of disorders, with a common genetic background (**Table 4**). A recent report examined 43 cases of pyoderma gangrenosum, acne and hidradenitis suppurativa (PASH) syndrome (136). Males appear to be more frequently affected than females, all patients present with acne, with an average age of onset of 17 years. Despite being the earliest symptom to manifest and primarily affecting the face or the face and trunk, features of acne are infrequently reported in literature. However, the frequency of nodulocystic lesions produces an acne phenotype quite similar to PAPA syndrome, despite acne fulminans is most commonly documented (136). In addition to acne, patients may also have papulopustular lesions, abscesses, and fistulae developing in draining sinuses and scars (137). PASH patients show a heterogeneous genetic background, and *PSTPIP1* abnormalities in the number of CCTG microsatellites in the *PSTPIP1* promoter region have been reported in a minority of cases. Otherwise, also different mutations in other AIDs-related genes or no genetic abnormalities have been described in PASH patients (136). Given the wide variability of PASH syndrome’s genetic background, PG, and its syndromic form PASH may constitute a spectrum of polygenic autoinflammatory disorders, as supported by the overexpression of proinflammatory cytokines IL-1β, IL-17, and TNFα, in affected patients (137). PG, acne, seronegative spondyloarthritis, with or without hidradenitis suppurativa (PASS) syndrome is a recently described PAID. Notably, all the few cases reported in literature were males, often with a nodulocystic acne spread to the face and trunk (138). A variant of unknown significance (VUS) of the *NLRP3* gene was discovered in a 20-year-old boy who had severe axillary and perineal SH, bilateral sacroiliitis, and PG (139). In a young PASS patient, the IL-17 inhibitor secukinumab was reported to be effective (140). Pyogenic arthritis, PG, acne, and hidradenitis suppurativa (PAPASH) syndrome is another different PAID. Two *PSTPIP1* gene mutations and one *MEFV* variant have been documented in PAPASH patients to date (141–143). PsAPASH is a different clinical form of PASH that includes psoriatic arthritis (PsA), with an unidentified underlying genetic background. It was first described in a 50-year-old male patient with PASH features and PsA (144). A 33-year-old male patient with nodulocystic acne was diagnosed with PG, acne, and ulcerative colitis (PAC) syndrome. Two years following the onset of the inflammatory colitis, pustulosis broke out, and a VUS of the *PSTPIP1* gene was detected. However, because it affected a gene’s highly conserved area, harmful consequences are likely (145). An uncommon and distinctive clinical PAID has been named as *PSTPIP1*-associated myeloid-related proteinaemia inflammatory (PAMI) syndrome. More than 35 cases of PAMI syndrome have been reported so far, mostly with childhood onset, and one of them dramatically improved after a combining therapy with prednisone and doxycycline for three months. Heterozygous variants of *PTPSTP1* are the most common pathogenic variants reported in PAMI syndrome (146). Its diagnosis is based on the results of DNA sequencing, the clinical features of typical lesions, leukopenia, and the significantly elevated serum calprotectin levels. However, high levels of this blood protein can be utilized as a screening indicator in suspected instances and are a characteristic of both PAPA and PAMI syndromes (146). Treating acne is frequently a main objective in PAIDs, and systemic medications are widely used to control other different symptoms, such as pyogenic arthritis, and inflammatory colitis. Isotretinoin is frequently the first treatment option for acne, although some patients do not entirely respond and require additional medications (136, 147). Current reports mostly focus on IL-1 inhibitors, and TNFα antagonists, with reported good results (145, 148–150). Indeed, traditional approaches using other anti-inflammatory drugs are often ineffective, and dual therapies rarely reported (147, 150). According to a recent review of the literature, anakinra, glucocorticoids, infliximab, and adalimumab generally produce good response in PAIDs, whereas nonsteroidal anti-inflammatory drugs seem to be only partially beneficial on moderate phenotypes of PAPA syndrome (147). However, clinical improvement can vary significantly from patient to patient, and due to the low prevalence of PAPA syndrome and PAIDs, no therapeutic strategies based on guidelines are available (136, 151).

**Table 4:** *PSTPIP1*-associated autoinflammatory diseases.

Acronym	Complete disease name
PAPA	Pyogenic arthritis, pyoderma gangrenosum and acne
PASH	Pyoderma gangrenosum, acne and hidradenitis suppurativa
PAPASH	Pyogenic arthritis, pyoderma gangrenosum, acne, and hidradenitis suppurativa
PsAPASH	Pyogenic arthritis, pyoderma gangrenosum, acne, hidradenitis suppurativa and psoriatic arthritis
PASS	Pyoderma gangrenosum, acne, seronegative spondyloarthritis, with or without hidradenitis suppurativa
PAMI	*PSTPIP1*-associated myeloid-related proteinaemia inflammatory
PAC	Pyoderma gangrenosum, acne, and ulcerative colitis

### 6.5 Periodic fever, immunodeficiency and thrombocytopenia syndrome

Infancy-onset periodic fever, recurring respiratory infections, and thrombocytopenia are common features of PFIT. Even though severe viral illnesses, such as varicella, have been recorded, recurrent bacterial infections are more commonly reported (65). In addition to the classical phenotype, PFIT patients may also present with skin and mucosal ulcerations, aphthous stomatitis, nodulocystic acne, bronchiectasis, and poor growth. Leukocytosis, increased neutrophils, and elevated ferritin, but also lymphopenia, mild neutropenia, and anemia have been described during autoinflammatory attacks (65–67). Although bone marrow transplantation may be effective, prognosis is usually not favourable (65–67).

## 7 Conclusions

Significant progress has been made in comprehending pyrin’s role in AIDs, but a great deal of research is still required. The growing knowledge about RhoA GTPase, PKN1/2, and gasdermin D functions, as well as the discovery of the role of alarmins S100A8/A9 and S100A12 in the inflammatory cascade, are allowing for a better understanding of the molecular mechanisms behind pyrin. In addition, the interaction of pyrin with the cytoskeleton and the description of new pyrin-related disorders in detail are leading to a greater comprehension of these immune pathways. A significant number of pathophysiological processes, such as the mechanisms by which pyrin is dephosphorylated through the release of 14-3-3, still require further investigation, and there are still some areas of uncertainty. Additional evidence is needed to comprehend the mechanisms underlying the efficacy of colchicine therapy and the precise mechanisms behind the B30.2/SPRY domain. Lastly, standardized and more effective treatment strategies for several pyrin-related AIDs have yet to be validated, although treatment with IL-1 inhibitors has often demonstrated good efficacy and safety.

## Author contributions

SLB: Conceptualization, Investigation, Visualization, Writing – original draft. AD: Investigation, Visualization, Writing – original draft. GD: Investigation, Visualization, Writing – original draft. OB: Resources, Supervision, Validation, Writing – review & editing. SO: Resources, Supervision, Visualization, Writing – review & editing. MG: Supervision, Validation, Visualization, Writing – review & editing. FC: Supervision, Validation, Visualization, Writing – review & editing. LB: Supervision, Validation, Visualization, Writing – review & editing.

## Glossary

**Table:**

(AIDs)	Autoinflammatory diseases
(*ARPC1B*)	actin-related protein 2/3 complex subunit 1B
(ASC)	apoptosis associated speck-like protein containing CARD
(CARD)	caspase activation and recruitment domain
(CC)	coiled-coil
(CD-BP1)	CD2-binding protein 1;(crFMF), Colchicine-resistant
(DAMPs)	danger-associated molecular patterns
(ELE)	erysipelas like erythema
(EMA)	European medicines agency
(FDA)	food and drug administration
(FMF)	familial Mediterranean fever
(GSDMD)	gasdermin D
(HAMPs)	homeostasis-altering molecular processes
(HIDS)	hyperimmunoglobilinemia D with periodic fever syndrome
(IL)	interleukin
(MA)	mevalonic aciduria
(MVK)	mevalonate kinase
(MKD)	mevalonate kinase deficiency
(NOCARH)	neonatal onset cytopenia with autoinflammation, cytopenia, rash, and hemophagocytes
(PAAND)	pyrin-associated autoinflammation with neutrophilic dermatosis
(PAC)	Pyoderma gangrenosum, acne, and ulcerative colitis
(PAMI)	*PSTPIP1*-associated myeloid-related proteinaemia inflammatory
(PAMPs)	pathogen-associated molecular patterns
(PAPA)	Pyogenic arthritis, pyoderma gangrenosum and acne
(PAPASH)	Pyogenic arthritis, pyoderma gangrenosum, acne, and hidradenitis suppurativa
(PASH)	Pyoderma gangrenosum, acne and hidradenitis suppurativa
(PASS)	Pyoderma gangrenosum, acne, seronegative spondyloarthritis, with or without hidradenitis suppurativa
(PFIT)	Periodic Fever Immunodeficiency and Thrombocytopenia
(PK)	porokeratosis
(PsAPASH)	Pyogenic arthritis, pyoderma gangrenosum, acne, hidradenitis suppurativa and psoriatic arthritis
(PRINTO)	paediatric rheumatology international trials organization
(PRRs)	pattern recognition receptors
(PAIDs)	*PSTPIP1*-associated inflammatory diseases
(NOD)-like receptors	nucleotide-binding oligomerization domain
(RhoA)	Ras homolog family member A
(SAA)	serum amyloid A
(TLRs)	toll-like receptors
(TNF)	tumor necrosis factor
(WDR1)	WD repeat-containing protein 1.
